# Approach, avoidance, and their conflict: the problem of anchoring

**DOI:** 10.3389/fnsys.2014.00124

**Published:** 2014-07-03

**Authors:** Neil McNaughton, Philip J. Corr

**Affiliations:** ^1^Department of Psychology and Brain Health Research Centre, University of OtagoDunedin, New Zealand; ^2^Department of Psychology, City University LondonLondon, UK

**Keywords:** approach, avoidance, conflict, personality, biomarkers, anchoring

To understand the neurobiology of individual differences in approach and avoidance behavior, we must anchor constructs at the behavioral level to the long-term global sensitivities of the neural systems that give rise to the observed stable patterns of behavior. We will argue that this requires not only appropriate data at both the neural and behavioral levels but also appropriate account to be taken of interactions at the intervening level of the conceptual nervous system (Hebb, 1949; Gray, 1975). In particular, in accounting for approach and avoidance behavior we must include consideration of the distinction between valuation and motivation (Corr and McNaughton, 2012), of interactions between the approach system and the avoidance system (Gray and Smith, 1969), and of their interaction with a distinct additional system that is activated by approach-avoidance conflict (Gray, 1977; summarized in Corr, 2013).

But first we need to ask why would we expect there to be traits linked to global approach and avoidance systems? Simple animals (with little or no brain) can produce approach and avoidance behavior (toward benefits and ultimately reproduction; and away from dangers and ultimately failure to reproduce) via multiple independent rules of thumb (Krebs et al., 1983). But we can expect more complex brains to have largely integrated these simple elements into systems more generally dedicated to approach or avoidance “because this is how [a few] genes can build a complex system that will produce appropriate but flexible behavior to increase fitness. … Rather than just pre-programmed movements such as tropisms and taxes, … if the genes are efficiently to control behavior … they must specify the goals for action.” (Rolls, 2000, pp. 183, 190). Together with the evolution of general approach and avoidance systems that are not tied to any specific motivating stimulus (reinforcer), we would expect evolution of the long-term adaptive control of their overall sensitivity to adequate inputs. Such stable sensitivity would be the neurobiological basis of approach and avoidance personality traits.

Determining the appropriate neurobiological measure for the sensitivity of a highly evolved approach or avoidance system is not simple. These systems have hierarchically organized neural levels with processing ranging from “quick and dirty” to “slow and sophisticated” for both perception (LeDoux, 1994) and action (Graeff, 1994, 2010). Sensitivity to input determines which level of the system is activated and so sensitivity cannot reside in any one of the modules within the system (McNaughton and Corr, 2004). The source of any sensitivity must, therefore, be identified independently—in essence requiring at least a preliminary surface level description of traits.

Existing theories of personality provide a number of competing surface level, lexically-derived, systems with trait measures that relate to approach and avoidance either indirectly via constructs such as Extraversion and Neuroticism (Eysenck, 1957) or directly via constructs such as Harm Avoidance (Cloninger et al., 1993). Each system is stable, with links to mental disorder (Strelau and Zawadzki, 2011; Gomez et al., 2012; Mullins-Sweatt and Lengel, 2012; Trull, 2012) and brain structure (Gardini et al., 2009; DeYoung et al., 2010). But even when starting with approach and avoidance as primary constructs, they are derived “top-down” from pools of lexically-chosen questionnaire items (Carver and White, 1994; Elliot and Thrash, 2010) not from biological anchors. They also depend on factor analysis, which determines the number of dimensions, *but not location of trait axes* of the personality “space” that items occupy (Lykken, 1971; Corr and McNaughton, 2008). It is little more than an act of faith to believe that the causal structure of personality is isomorphic with its lexical factor structure. So, even if we knew for certain that there were only two dimensions within a particular measured personality space, one questionnaire system could have a single simple trait anxiety dimension (orthogonal to, say, impulsiveness) that was a combination of neuroticism and introversion in another (Gray, 1970)—the two systems differing only on which items from an original pool were used to create scales. Factor analytically derived trait measures can also easily meet the criterion of having “simple structure” (in the sense that a set of items loads highly on only one factor so factors can be clearly identified by unique item loadings) while implying improbable causation (Lykken, 1971). Further, not only is there no reason to suppose that biologically accurate scales should have simple structure but also current scale systems, even though designed to have this, often do not (DeYoung, 2006, 2010).

The plethora of competing trait scales can to some extent be encompassed by just five major trait dimensions that include both normal people and those with psychiatric disorders (Markon et al., 2005; Revelle et al., 2011; Krueger et al., 2012). However, the traits of the competing systems have complex relations to these five large scale dimensions and it is open to question whether there are five fundamental dimensions or whether these are complex facets riding on two or even just one major dimension of personality (Markon et al., 2005; DeYoung et al., 2007; Rushton and Irwing, 2009). These large scale dimensions have “facets” that potentially represent the true underlying sources of personality; and different “approaches differ substantially in the number and nature of the facets they propose, indicating that further conceptual and empirical work is needed to achieve a consensual specification of the Big Five factors at lower levels of abstraction. [Further], given that the Big Five were derived initially from analyses of the personality lexicon, one might wonder whether they merely represent linguistic artifacts” (John et al., 2008, p 141). With no “bottom up” neural anchor to definitely locate the correct rotation of any true biological trait/facet axis, there is no unequivocal way to unify the various systems currently in use.

A related problem, on which we focus below, is that the bulk of personality research has required statistical independence (orthogonality) of the extracted factors. To do otherwise would greatly increase the already large number of alternative trait solutions for any particular item space. However, as we will see, there is good reason to see surface level behavior as being determined interactively even if the biological control of the underlying sensitivities is independent. Likewise, even if the control of factors is neurally independent, when one, e.g., neuroticism, is a risk factor for another, e.g., anxiety (Andrews et al., 1990), then they will become statistically linked in the population as a result.

The solution for approach/avoidance traits is to anchor their factor spaces to measures derived from existing neural state theory. Figure 1 is derived from one particular detailed neuropsychological theory (Gray and Smith, 1969; Gray, 1982; Gray and McNaughton, 2000; McNaughton and Corr, 2004; Corr and McNaughton, 2012) but its system level description captures issues that must be taken into account by any approach/avoidance account of personality. Adequate stimuli (reinforcers) must first be valued and, importantly, negative stimuli (e.g., losses) have a higher exchange rate that positive ones (e.g., gains); that is, people usually show loss aversion (Kahneman and Tversky, 1979).

**Figure 1:**
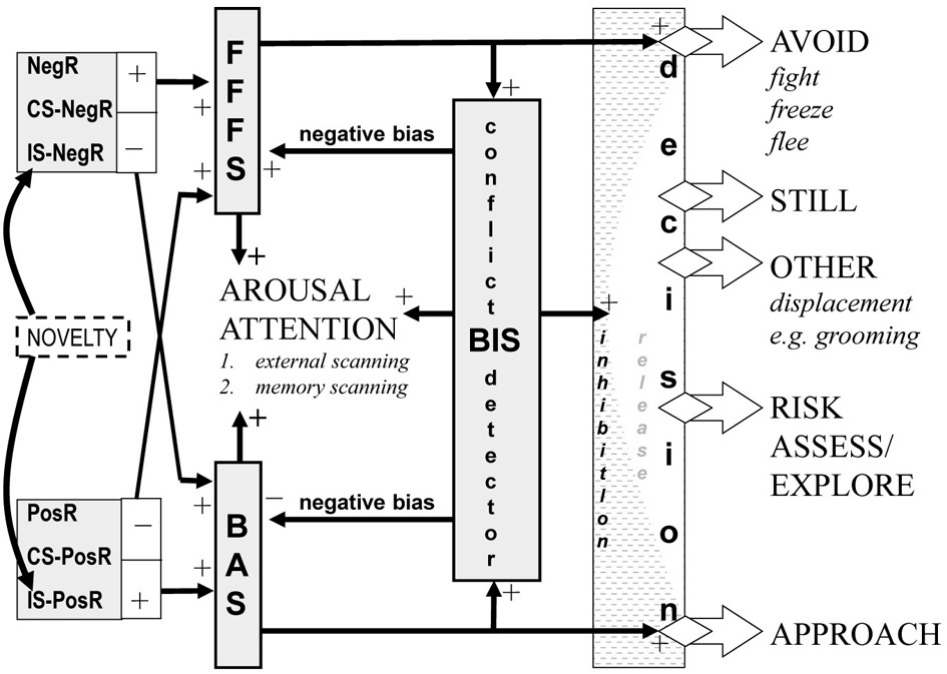
**Overall relation of approach (BAS), avoidance (FFFS = fight, freeze, flee), and conflict (BIS = behavioral inhibition) systems—an updated model**. The inputs to the system are classified in terms of the delivery (+) or omission (−) of primary positive reinforcers (PosR) or primary negative reinforcers (NegR) or conditional stimuli (CS) or innate stimuli (IS) that predict such primary events. The BIS is activated when it detects approach-avoidance conflict—suppressing prepotent responses and eliciting risk assessment and displacement behaviors. The systems interact in a variety of ways to generate behavior, see text. The shaded areas are all points at which traits appear to operate. Figure and legend modified from Gray and McNaughton (2000) and Corr and McNaughton (2012).

Any specific positive or negative reinforcer can produce approach or avoidance depending on its contingency (presentation or omission) with responding. For any given reinforcer, the motivational sensitivity of approach activation is different from avoidance activation; and these are separate from the distinct valuation sensitivities of gain to loss (Hall et al., 2011). The strength of response output for any given level of approach activation also depends on distance from the goal (not shown in Figure 1) and does so to a lesser extent than does avoidance (Miller, 1944).

Even with independent trait sensitivities, state approach output depends on the level of avoidance activation, and vice versa: their activations sum to generate arousal, while subtracting to determine choice—giving rise to phenomena such as behavioral contrast and peak shift (Gray and Smith, 1969). As a result, when approach and avoidance are strongly and equally activated, arousal is high but the probability of both approach and avoidance is low; in addition, the approach-avoidance conflict is detected by a third system (with its own trait sensitivity) that is unlike either pure approach or pure avoidance (withdrawal) in being affected by anxiolytic drugs (Gray, 1977). Both approach and avoidance are then inhibited and replaced by behaviors such as risk assessment (Gray and McNaughton, 2000) and displacement (Hinde, 1998), while arousal and negative bias (risk aversion) are increased. With this plethora of interactions, it will be difficult to extract true approach and avoidance traits from the surface structure of behavior—especially if orthogonal factors such as gain and approach have been conflated in a single construct such as reward (Corr and McNaughton, 2012).

However, neural measures should be able to target the internal representations of the specific elements depicted in Figure 1; challenge their response with appropriate combinations of stimuli; and so dissect out the specific contribution of a particular trait sensitivity. These neural measures can then be used to anchor traits within the conventional factor spaces and determine non-orthogonalities. Paradoxically, we are closest to achieving this with the most embedded neural construct: sensitivity to conflict. The argument for the use of primarily neural rather than questionnaire measures of approach and avoidance sensitivities has been made in detail previously—coupled with arguments for combining bottom up neural analysis with top down behavioral analysis (Smillie, 2008a,b; DeYoung, 2010). Here, we would emphasize, in addition, that the choice of neural measures should be strongly theoretically based and behaviorally and or pharmacologically validated in relation to the theory. Otherwise a plethora of questionnaires becomes a plethora of putative neural measures.

The conflict system is defined by the action of anxiolytic drugs (Gray, 1977) acting on receptors for endogenous compounds (Guidotti et al., 1978; Polc, 1995) that could mediate the system's trait sensitivity. Anxiolytic action is specifically linked to hippocampal rhythmicity in rodents (Woodnorth and McNaughton, 2002; McNaughton et al., 2006, 2007) and this has led to development of a human scalp EEG homolog (McNaughton et al., 2013) that provides a biomarker for conflict sensitivity in humans. This biomarker appears to be linked to the shared variance in neuroticism and trait anxiety much more than either of their unique variances (Neo et al., 2011).

In summary, we believe that approach and avoidance systems have evolved in such a way that global control of sensitivities to gain, loss, approach, avoidance and conflict can underlie human personality traits (Corr and McNaughton, 2012). While each of these long-term sensitivities is likely to be controlled independently, under normal ecological circumstances short-term behavioral output will be the result of complex interactions between them (Figure 1). However, the combination of appropriate neural measures with designs that dissect these interactions should provide the means to anchor trait measures in the data spaces that personality research has already shown have long term stability and important behavioral, and particularly psychiatric, consequences. Critically, the factor analysis of lexically-derived variables at the surface level of description cannot be assumed to reflect the deeper construct processes that are giving rise to surface descriptions; and no adjustment of the basic factor analysis method can avoid the problem created when there is no neural anchor to ensure inclusion of correct items and unique rotational solution after initial factoring.

## Conflict of interest statement

The authors declare that the research was conducted in the absence of any commercial or financial relationships that could be construed as a potential conflict of interest.
